# Supplementary material for: Quadrivalent Vaccines for the Immunization of Adults against Influenza: A Systematic Review of Randomized Controlled Trials
Source: Int J Environ Res Public Health. 2022 Aug 1;19(15):9425. doi: 10.3390/ijerph19159425 (PMC9368426; doi:10.3390/ijerph19159425)
Supplement: Supplementary file 1 [file ijerph-19-09425-s001.zip › ijerph-1819750-supplementary.pdf]

International Journal of
Environmental Research and Public Health

Supplementary Materials

Table S1. Medical subject headings (MeSH) and keyword used for the literature search.

MEDLINE	"influenza vaccines" [Mesh] AND (efficacy [title/abstract] OR immunogenicity [title/abstract])<br>*Filters applied: Randomized Controlled Trial, Adult: 19+ years*
COCHRANE	("influenza vaccine"): ti, ab, kw AND (efficacy OR immunogenicity): ti, ab, kw AND (adult): ti, ab, kw NOT (children): ti, ab, kw AND (trial): ti, ab, kw
SCOPUS	(TITLE-ABS-KEY ("influenza vaccines") AND TITLE-ABS-KEY ((efficacy OR immunogenicity) AND trial) AND TITLE-ABS-KEY (adult) AND NOT TITLE-ABS-KEY (children))

Table S2. Characteristics of included RCTs.

First Author, Year	Country	Study Population	Mean Age (SD)	% Male	N. Experimental Arm (Adult Population)	N. Control Arm (Adult Population)	Experimental/Control Arm	Endpoints
Block, 2011 [22]	USA	18–49 yo	Experimental: 32 *<br>Control: 32 *<br>Overall: 32 *	Experimental: 45.2<br>Control: 44.0<br>Overall: 44.8	1200	Control (Vic): 301<br>Control (Yam): 299	Q/LAIV/ T/LAIV	SCR, SCF
Beran, 2013 [23]	Czech Republic	18-60 yo	Experimental (QIV): 38.6 (11.82)<br>Experimental (LD QIV-AS): 37.7 (12.30)<br>Control (TIV): 37.4 (12.51)<br>Control (LD TIV-AS): 36.7 (12.84)<br>Overall: 37.6 (12.35)	Experimental (QIV): 42.3<br>Experimental (LD QIV-AS): 36.5<br>Control (TIV): 44.8<br>Control (LD TIV-AS): 36.5<br>Overall: 40.0	Experimental (QIV): 105<br>Experimental (LD QIV-AS): 105	Control (TIV): 105<br>Control (LD TIV-AS): 105	QIV, LD QIV-AS/TIV, LD TIV-AS	SCR, SPR, SCF
Greenberg, 2013 [24]	USA	≥18 yo	Experimental: 56.7 (17.2)<br>Control (Vic): 55.0 (18.4)<br>Control (Yam): 54.9 (17.5)	Experimental: 32%<br>Control (Vic): 35%<br>Control (Yam): 32%	190	Control (Vic): 190<br>Control (Yam): 190	QIV/TIV	SCR, SPR, SCF
Kieninger, 2013 [25]	Germany, Romania, Spain, Korea, USA	≥18 yo	Experimental: 57.9 (17.7)<br>Control (Vic): 58.1 (17.8)<br>Control (Yam): 58.1 (17.9)	Experimental: 42.5%<br>Control (Vic): 45.7%<br>Control (Yam): 43.8%	3036 ‡	Control (Vic): 1010 ‡<br>Control (Yam): 610 ‡	QIV/TIV	SCR, SPR, SCF
Pépin, 2013 [26]	France, Germany	≥18 yo	Experimental: 55.1 (16.8)<br>Control (Vic): 55.1 (16.9)<br>Control (Yam): 56.0 (16.3)	Experimental: 41.1%<br>Control (Vic): 47.8%<br>Control (Yam): 37.7%	1116	Control (Vic): 226<br>Control (Yam): 223	QIV/TIV	SCR, SPR, SCF
Sheldon, 2013 [27]	USA	18–49 yo	Experimental: 33.9 (9.5)<br>Control (Vic): 33.8 (8.8)<br>Control (Yam): 34.0 (9.1)<br>Overall: 33.9 (9.3)	Experimental: 41.7%<br>Control (Vic): 44.0%<br>Control (Yam): 43.7%<br>Overall: 42.4%	1202	Control (Vic): 298<br>Control (Yam): 300	Q/LAIV/T/LAIV	SCR, SPR
Tinoco, 2014 [28]	Canada, Mexico, USA	≥18 yo	Experimental: 50.0 (19.5)<br>Control (Vic): 50.8 (18.5)<br>Control (Yam): 49.6 (19.3)	Experimental: 38.6%<br>Control (Vic): 41.3%<br>Control (Yam): 36.7%	1272 ‡	Control (Vic): 213 ‡<br>Control (Yam): 218 ‡	QIV/TIV	SCR, SPR
Cadorna-Carlos, 2015 [29]	Australia, Philippines	9-60 yo	Experimental: 38.0 (12.4) †<br>Control: 36.3 (12.4) †	Experimental: 38.6% †<br>Control: 51.8% †	1649	56	QIV/TIV	SCR, SPR, SCF
Gorse, 2015 [30]	USA	18–64 yo	Experimental: 41.6 (13.2)<br>Control (Vic): 41.9 (13.3)<br>Control (Yam): 41.2 (13.5)	Experimental: 38.9%<br>Control (Vic): 37.6%<br>Control (Yam): 39.7%	1676	Control (Vic): 847<br>Control (Yam): 837	IIV4-ID/IIV3-ID	SCR, SPR
Bart, 2016 [31]	USA	≥18 yo	Experimental: 57.4 (17.8)<br>Control (Vic): 57.1 (18.1)<br>Control (Yam): 57.2 (18.0)	Experimental: 45.2%<br>Control (Vic): 41.4%<br>Control (Yam): 42.0%	674	Control (Vic): 332<br>Control (Yam): 334	cQIV/cTIV	SCR, SPR, SCF

Pillet, 2016 [32]	Canada	18-49 yo	Experimental (3 µg): 33.5 (10.58)<br>Experimental (9 µg): 34.9 (8.32)<br>Experimental (15 µg): 31.9 (9.67)<br>Control: 34.4 (9.04)	Experimental (3 µg): 50.0%<br>Experimental (9 µg): 33.3%<br>Experimental (15 µg): 60.0%<br>Control: 46.7%	Experimental (3 µg): 30<br>Experimental (9 µg): 30<br>Experimental (15 µg): 30	30	QVLP/placebo	SCR, SPR, SCF
Choi, 2017 [33]	Republic of Korea	≥19 yo	Experimental: 43.36 (14.46)<br>Control (Vic): 42.96 (14.14)<br>Control (Yam): 43.38 (14.20)<br>Overall: 43.27 (14.31)	Experimental: 36.7%<br>Control (Vic): 38.4%<br>Control (Yam): 36.2%<br>All: 37%	599	Control (Vic): 305<br>Control (Yam): 299	cQIV/cTIV	SCR, SPR, SCF
Dunkle, 2017 [34]	USA	18-49 yo	Experimental: 33.3 (N.A.)<br>Control: 33.0 (N.A.)	Experimental: 36%<br>Control: 33%	1011	339	RIV4/QIV	SCR
Dunkle, 2017 [35]	USA	≥50 yo	Experimental: 63 (N.A.)<br>Control: 63 (N.A.)	Experimental: 41.5%<br>Control: 41.6%	2569	2617	RIV4/QIV	Laboratory-confirmed influenza, SCR, SPR
Treanor, 2017 [36]	USA	≥18 yo	Experimental: 58.3 (18.10)<br>Control (Vic): 58.3 (17.89)<br>Control (Yam): 58.2 (18.10)<br>Overall: 58.3 (18.04)	Experimental: 44.2%<br>Control (Vic): 41.5%<br>Control (Yam): 41.3%<br>Overall: 42.8%	1741	Control (Vic): 872<br>Control (Yam): 871	QIV/TIV	SCR, SPR
Wang, 2017 [37]	China	≥3 yo	Experimental: 44.49 (10.81) †<br>Control (Vic): 45.33 (11.42) †<br>Control (Yam): 45.06 (10.83) †	Experimental: 44.66% †<br>Control (Vic): 44.59% †<br>Control (Yam): 44.98% †	458	Control (Vic): 231<br>Control (Yam): 229	QIV/TIV	SCR, SPR, SCF
Choi, 2018 [38]	Republic of Korea	18-60 yo	Experimental: 35.9 (9.1)<br>Control: 35.6 (9.5)	Experimental: 25%<br>Control: 29%	200	100	QIV/TIV	SCR, SPR, SCF
Sesay, 2018 [39]	France, Poland, Germany, Belgium	≥18 yo	Experimental (lot 1): 54.5 (18.1)<br>Experimental (lot 2): 54.8 (18.2)<br>Experimental (lot 3): 54.5 (18.1)<br>Control (Vic): 55.6 (16.9)<br>Control (Yam): 53.7 (18.6)	Experimental1 (lot 1): 44.1%<br>Experimental2 (lot 2): 45.2%<br>Experimental3 (lot 3): 49.5%<br>Control (Vic): 45.3%<br>Control (Yam): 45.1%	Experimental1 (lot 1): 554<br>Experimental2 (lot 2): 555<br>Experimental3 (lot 3): 561	Control (Vic): 279<br>Control (Yam): 276	QIV/TIV	SCR, SPR, SCF
Sharma, 2018 [40]	India	≥18 yo	Experimental: 45.7 (19.4)<br>Control: 44.8 (18.6)	with cohort >60<br>Experimental: 56.2%<br>Control: 62.1%	176	174	QIV/TIV	SCR, SPR
van de Witte, 2018 [41]	Belgium, Germany, Hungary, Latvia, Lithuania	≥18 yo	Experimental: 55.9 (17.6)<br>Control (Vic): 55.4 (18.0)<br>Control (Yam): 55.0 (17.6)<br>All: 55.7 (17.7)	Experimental: 43.2%<br>Control (Vic): 45.2%<br>Control (Yam): 43.0%<br>All: 43.4%	1538	Control (Vic): 221<br>Control (Yam): 221	QIV/TIV	SCR, SPR, SCF
Pillet, 2019 [42]	Canada	18-49 yo	Experimental (15 µg): 33.9 (9.0)<br>Experimental (30 µg): 32.5 (8.93)<br>Experimental (60 µg): 43.5 (8.73)<br>Control: 35.8 (9.38)	Experimental1 (15 µg): 44%<br>Experimental2 (30 µg): 41.3%<br>Experimental3 (60 µg):	Experimental (15 µg): 75<br>Experimental (30 µg): 75<br>Experimental (60 µg): 75	75	QVLP/placebo	SCR, SPR

Song, 2019 [43]	Republic of Korea	≥19 yo	Experimental: 40.6 (14.1)<br>Control (Vic): 41.0 (14.8)<br>Control (Yam): 40.0 (14.8)	46.7%<br>Control: 42.7<br>Experimental: 37.7%<br>Control (Vic): 40.0%<br>Control (Yam): 38.2%	583	Control (Vic): 291<br>Control (Yam): 292	QIV/TIV	SCR, SPR
Vesikari, 2020 [44]	Finland	Pregnant women ≥18 yo at 20 to 32 week of pregnancy	Experimental: 32.1 *<br>Control: 30.7 *	0	230	116	QIV/TIV	SCR, SPR, SCF
Ward, 2020 [45]	Canada, USA, Finland, Germany, UK, Philippines, Thailand	18-64 yo	Experimental: 44.4 (13.81)<br>Control: 44.7 (13.62)	Experimental: 39.8%<br>Control: 40.2%	5077	5083	QVLP/placebo	Laboratory-confirmed influenza, SCR, SPR

SD: standard deviation; * Median; yo: years old; N.A.: not available; † data referred to 18-60 year old population; ‡ data referred to ≥18 year old population; Vic: Victoria B lineage; Yam: Yamagata B lineage; SCR: seroconversion rates; SPR: seroprotection rates; SCF: seroconversion factors; Q/LAIV/: quadrivalent live attenuated influenza vaccine; T/LAIV: trivalent live attenuated influenza vaccine; QIV: standard quadrivalent influenza vaccine; TIV: trivalent quadrivalent influenza vaccine; LD QIV-AS: low-dose adjuvanted quadrivalent influenza vaccine; LD TIV-AS: low-dose adjuvanted trivalent influenza vaccine; IIV4-ID: quadrivalent intradermal influenza vaccine; IIV3-ID: trivalent intradermal influenza vaccine; cQIV: cell culture-based, inactivated, quadrivalent influenza vaccine; cTIV: cell culture-based, inactivated, trivalent influenza vaccine; QVLP: plant derived quadrivalent vaccine RIV4: quadrivalent recombinant influenza vaccine.
